# Diagnostic Value of the Fimbriae Distribution Pattern in Localization of Urinary Tract Infection

**DOI:** 10.3389/fmed.2021.602691

**Published:** 2021-06-18

**Authors:** Xiao Li, Kaichen Zhou, Jingyu Wang, Jiahe Guo, Yang Cao, Jie Ren, Tao Guan, Wenchao Sheng, Mingyao Zhang, Zhi Yao, Quan Wang

**Affiliations:** ^1^Key Laboratory of Immune Microenvironment and Disease of the Educational Ministry of China, Tianjin Key Laboratory of Cellular and Molecular Immunology, Department of Immunology, School of Basic Medical Sciences, Tianjin Medical University, Tianjin, China; ^2^Xuzhou Key Laboratory of Laboratory Diagnostics, School of Medical Technology, Xuzhou Medical University, Xuzhou, China; ^3^Department of Clinical Laboratory, The Second Hospital of Tianjin Medical University, Tianjin, China; ^4^China Unicom Software Research Institute, Xi'an, China; ^5^2011 Collaborative Innovation Center of Tianjin for Medical Epigenetics, Tianjin Medical University, Tianjin, China

**Keywords:** upper urinary tract infections, lower urinary tract infections, UPEC, fimbriae, machine learning, XGBoost

## Abstract

Urinary tract infections (UTIs) are one of the most common infectious diseases. UTIs are mainly caused by uropathogenic *Escherichia coli* (UPEC), and are either upper or lower according to the infection site. Fimbriae are necessary for UPEC to adhere to the host uroepithelium, and are abundant and diverse in UPEC strains. Although great progress has been made in determining the roles of different types of fimbriae in UPEC colonization, the contributions of multiple fimbriae to site-specific attachment also need to be considered. Therefore, the distribution patterns of 22 fimbrial genes in 90 UPEC strains from patients diagnosed with upper or lower UTIs were analyzed using PCR. The distribution patterns correlated with the infection sites, an XGBoost model with a mean accuracy of 83.33% and a mean area under the curve (AUC) of the receiver operating characteristic (ROC) of 0.92 demonstrated that fimbrial gene distribution patterns could predict the localization of upper and lower UTIs.

## Introduction

Urinary tract infections (UTIs) are one of the most common infectious diseases (1), and are predominantly caused by uropathogenic *Escherichia coli* (UPEC) (2). UTIs are classified as either upper (pyelonephritis and ureteritis) or lower (cystitis and urethritis) according to the infection site (3–5). Lower UTIs usually induce cystitis and can progress into upper UTIs, resulting in pyelonephritis and ultimately renal failure. A urinalysis positive for elevated leukocytes and a urinary culture positive for bacteria reinforce the clinical diagnosis of a UTI (6). The clinical symptoms are commonly regarded as the standard to differentiate the site of infections (7). UTIs are usually treated with antibiotics (8). Because of many factors such as underlying diseases, status of patient, antibiotic susceptibility, medication history, and infection sites (9), the therapies and medications used for upper and lower UTIs are different, so reasonable and accurate antibiotic use seems particularly important in the clinical treatment.

Machine learning is the core of artificial intelligence (AI) and the fundamental way to make computers intelligent. Artificial intelligence algorithms can go beyond human reasoning and build diagnostic models from a series of complex combinations to provide a more sensitive tool to discriminate among different conditions. In addition to successful application in anatomical (10) and functional imaging (11), machine learning techniques have also been successfully applied to identify bacterial species to differentiate the microbiomes of elders with Alzheimer's disease from those without dementia (12) and to predict personalized glycemic response to exercise by integrating baseline microbial signatures (13). When deep learning approaches were applied to bacterial Raman spectra, 30 common clinically relevant bacterial pathogens and their empiric treatments were accurately identified (14). The combination of LC-MS/MS and machine learning allows rapid and specific identification of 15 bacterial species representing 84% of all urinary tract infections (15). Furthermore, machine learning algorithms combined with a large dataset accurately diagnosed positive urine culture results (16).

UPEC express a variety of virulence factors, including fimbriae, toxins, iron-acquisition systems, metabolic enzymes, flagella, and surface polysaccharide structures (17, 18). Fimbriae are expressed on the bacterial surface and mediate several biological functions, including adhesion, invasion, and biofilm formation. Several types of fimbriae have been described in gram-positive and gram-negative bacteria (19). In gram-negative bacteria, the chaperone-usher (CU) fimbriae are most abundant. Thirty-eight distinct CU fimbrial operons have been identified in *E. coli* genomes, and 22 fimbrial gene clusters are distributed among the analyzed UPEC strains, with eight to 13 different gene clusters in a single UPEC strain (20). UPEC use a variety of fimbriae to adhere to the urethral epithelium, thereby promoting colonization and exerting its virulence in site-specific UTIs. For example, type 1 fimbriae, encoded by the *fim* operon, mediate UPEC colonization and invasion of bladder epithelial cells (21, 22), targeted therapeutic small inhibitor molecules and vaccines have fast-acting efficacy in treating UTIs in preclinical murine models (23–25). F9 fimbriae recognize uromodulin on the surface of the bladder epithelium through adhesin FmlH and participated in the occurrence of chronic cystitis (26). Yad fimbrial adhesin YadC promotes acute cystitis by interacting with the receptor ANXA2 on the bladder epithelium (27). In addition, P fimbriae are associated with acute pyelonephritis (28). Ygi fimbriae are necessary for adhesion to kidney epithelium, biofilm formation and *in vivo* fitness in kidneys (29). In most previous studies, the importance of a single type of fimbriae in lower and upper UTI pathogenesis is demonstrated. However, few studies have examined the combined effect of multiple fimbriae. Notably, deletion of both type 1 and F17-like fimbriae in a single strain produced lower intestinal fitness than either individual deletion (24), suggesting that each type of fimbriae has a different function in adherence or binding to a different site. Due to findings that different fimbriae can be used to type UPEC strains (30–32), this work sought to determine whether combined multiple fimbrial genes of UPEC could be used as targets to identify the localization of upper and lower UTIs.

Most predictive models for UTIs use a few variables such as urine dipstick or urinalysis results (33, 34), however, fimbriae have not been used in the diagnosis of UTI localization. Therefore, in this study, to explore the value of UPEC fimbriae in the diagnosis of infection sites, the distribution patterns of 22 fimbriae genes in clinically isolated UPEC strains and their relations to UTI localization were analyzed. The distribution patterns of the 22 fimbrial genes were correlated with the infection sites, demonstrating predictive value for the localization of upper and lower UTIs. Thus, a new method to identify the site of UTIs was created with innovative use of machine learning regarding UPEC fimbriae.

## Materials and Methods

### Bacterial Strains

A total of 144 UPEC strains were collected from patients diagnosed with UTIs at the Clinical Microbiology Laboratory of the Second Hospital of Tianjin Medical University (Tianjin, China) from 2014 to 2019. The laboratory criteria for infection were that the growth of a single *E. coli* strain in number >10^5^ CFU/mL or between 10^3^ and 10^5^ CFU/mL with >30 white blood cells/field in centrifuged urine. The strains were isolated from adult patients who presented with clinical syndromes, and the clinical information about the patients was obtained by review of their medical records. The studies involving human participants were reviewed and approved by the Ethics Committee of Tianjin Medical University. The patients provided their written informed consent to participate in this study.

The lower UTI group included 85 patients with acute uncomplicated cystitis. Diagnostic criteria included dysuria, urgency, and frequency, with or without suprapubic pain and hematuria; absence of flank pain; and fever <38°C.The upper UTI group included 59 patients with acute uncomplicated pyelonephritis. Diagnostic criteria included fever >38°C and flank pain, usually accompanied by lower urinary symptoms and sometimes by nausea, vomiting, and chills. The urine specimens of patients were obtained by clean catch voided midstream urine. Urine samples were cultured at 37°C in 5% CO_2_ overnight on blood agar plates (Hopebio, Qingdao, China), and then, a single colony was picked and identified using a VITEK MS full-braking microbial mass spectrometry detection system (bioMérieux, Inc., France). All UPEC strains used in this study are listed in Supplementary Table 1. The UPEC strains were cultured at 37°C overnight in Luria-Bertani broth.

### DNA Extraction

Two milliliters of the overnight bacterial cultures were collected, and the bacterial genome was extracted using a TIANamp Bacteria DNA kit (DP302, Tiangen, Beijing, China). The extracted genome was dissolved in sterile water, and DNA concentration was measured using a Nanodrop spectrophotometer (Thermo Fisher, Waltham, MA, USA). The DNA was frozen at −20°C until use.

### Gene Amplification

The genes encoding the usher proteins were amplified using the primers listed in Table 1, as previously described (31). A total of 30 μL of reaction mixture was used, and the end-point PCR setup was the following: denaturation at 95°C for 50 s, annealing for 45 s, extension at 72°C for 1 min, and a final extension at 72°C for 5 min. The annealing temperatures were different for the 22 genes, as shown in Table 1.

**Table 1:** Primers for PCR.

Primer^*^	**Direction**	**Sequence (5^**′**^ to 3^**′**^)**	**Tm (^**°**^C)**	**Amplicon size (bp)**	
CS1-like	Forward	GCTTGTACAACCGACAACA	51	755	a
	Reverse	CTCTGTTCATCCTGTTCAGA			
Mat	Forward	ATGGACAGTTACGCATCC	50	745	a
	Reverse	TCCACATCGTAAATACCGTA			
Type1	Forward	ATGCCGCAGGTAATAGTG	50	680	a
	Reverse	GAATTGCTCATCGACATTAC			
F1C/S	Forward	CGATTGTACCTGACCGTTCCT	59	654	This study
	Reverse	CAGATGCCCTTCACGTTGC			
F9	Forward	CGACACTTGCAGATGACAC	51	536	a
	Reverse	TGACATACTGTAACTGGCGT			
Ycb	Forward	GTTGAGATAACGCCAGAGA	51	727	a
	Reverse	CACTCGACGACGTAGAGTAG			
Auf	Forward	CTTTCGGTAACTACGGGTCT	54	838	This study
	Reverse	CTGGCTGTAGCACCGAAT			
Sfm	Forward	ATTAGAGAATGGCACATCC	54	862	a
	Reverse	ATCGCCATTTGAAGATGT			
LPF	Forward	AATAGTTACGCCACCTATTC	49	550	a
	Reverse	TGAAGAGTACGCGATAGC			
ECSF-0165	Forward	CTCCGTGAGTTCGGTCTT	52	813	a
	Reverse	AACAGGTGTCTCAGCATGAT			
ECSF-4008	Forward	CTGATGGTGATAATGCCA	53	1,008	a
	Reverse	ACTGAGGCTCAGACACACTA			
CS12	Forward	ATGTCTCGCGTCAATGTC	54	730	a
	Reverse	CAGCATCGTAATAGTGTTCA			
AFA	Forward	GTACCTGAAGTACAACGTCAC	53	543	a
	Reverse	CAGGACGTACTGTATGACG			
Yeh	Forward	CAGGTCGTAGCCATATTGA	52	607	a
	Reverse	TGATTCTCGTCATAAGCATG			
Yeh-like	Forward	CTGCCTAAGGTGCTACTAAC	55	688	a
	Reverse	TGCTGACATCGAGATCAGA			
F17-like	Forward	GTCATGGTAACCCTGTGC	51	529	a
	Reverse	GCAAGGTCATGCATTATACT			
Yfc	Forward	TCGCAACATGAGCATCTC	53	667	a
	Reverse	GTAGCTACCGTCACGCAA			
P	Forward	CCACCCAGACTGCGAGGCTAT	64	546	This study
	Reverse	GTCGGCATCCGCATTATCAAA			
Pix	Forward	GCTGTACACCGTCACACTC	53	812	a
	Reverse	TATCAGACATCCGCAACA			
Yad	Forward	AGCCATGCTTTCCTACAACC	56	564	This study
	Reverse	ATATCCCAGCGACCAACG			
Yqi	Forward	CCGCAACATCTCCTACAG	52	757	a
	Reverse	CGCGCTTTCACTAATGTT			
Ybg	Forward	ACCAAATCAGTAACGGACA	51	451	a
	Reverse	CCTGACTGTTCATGGTTATC			

* *The primers for PCR are based on the sequences of usher protein encoding genes*.

*^a^These primers were used as described by another study (31), others were designed in this study*.

### Cluster Analysis

The presence or absence of each of the 22 fimbrial genes in each strain was confirmed by PCR, and “presence” and “absence” were scored as “1” and “0,” respectively. A binary matrix was then constructed to investigate whether the distribution patterns of the 22 fimbrial genes were associated with the infection site. Clusters were formed sequentially by starting with the most similar pair of objects and forming higher clusters step-by-step. Then, squared Euclidean distances were used to calculate the difference and Ward's method was used to evaluate the distances between the clusters (35). The cluster analysis was visualized with a dendrogram.

### XGBoost

XGBoost (extreme gradient boosting), an implementation of gradient boosting machines, has recently been a dominating algorithm for its ability to model non-linear associations and general acceptance in machine learning (16, 36, 37). Measures including model training and evaluation were performed on Python 3.8.1 with xgboost package. The area under the curve (AUC) of the receiver operating characteristic (ROC) was used as the primary measure of model prediction (38). Diagnostic accuracy was defined as the proportion of all tests that gave a correct result. Five-fold cross-validation was conducted to evaluate the classifier performance, which was the process of rearranging the data so as to ensure that each fold was a good representative of the whole. Briefly, all 90 strains were divided into five equal parts, one of which was used as the validation set and the other four as the training set. Then, each fold was regarded once as the validation set, with the other four parts as the training set; after repeating the process five times, the average value was obtained. The AUC and ROC curve for each fold are presented, with the average AUC and ROC curve shown in black. A permutation test (*n* = 10,000) was used to determine the statistical significance of the model. The ranking of the real test statistic among the shuffled test statistics determined the *p*-value.

### Statistical Analyses

Descriptive statistics were used for outcomes. To analyze the ratios of different fimbrial genes and the relations with UTI infection sites, chi-square and Fisher tests were used. To account for multiple comparisons, a Bonferroni adjusted *p*-value (0.05/22 = 0.00227) was used to indicate statistical significance.

## Results

### Distribution Patterns of 22 Fimbrial Genes Were Correlated With Infection Site

**Figure 1:**
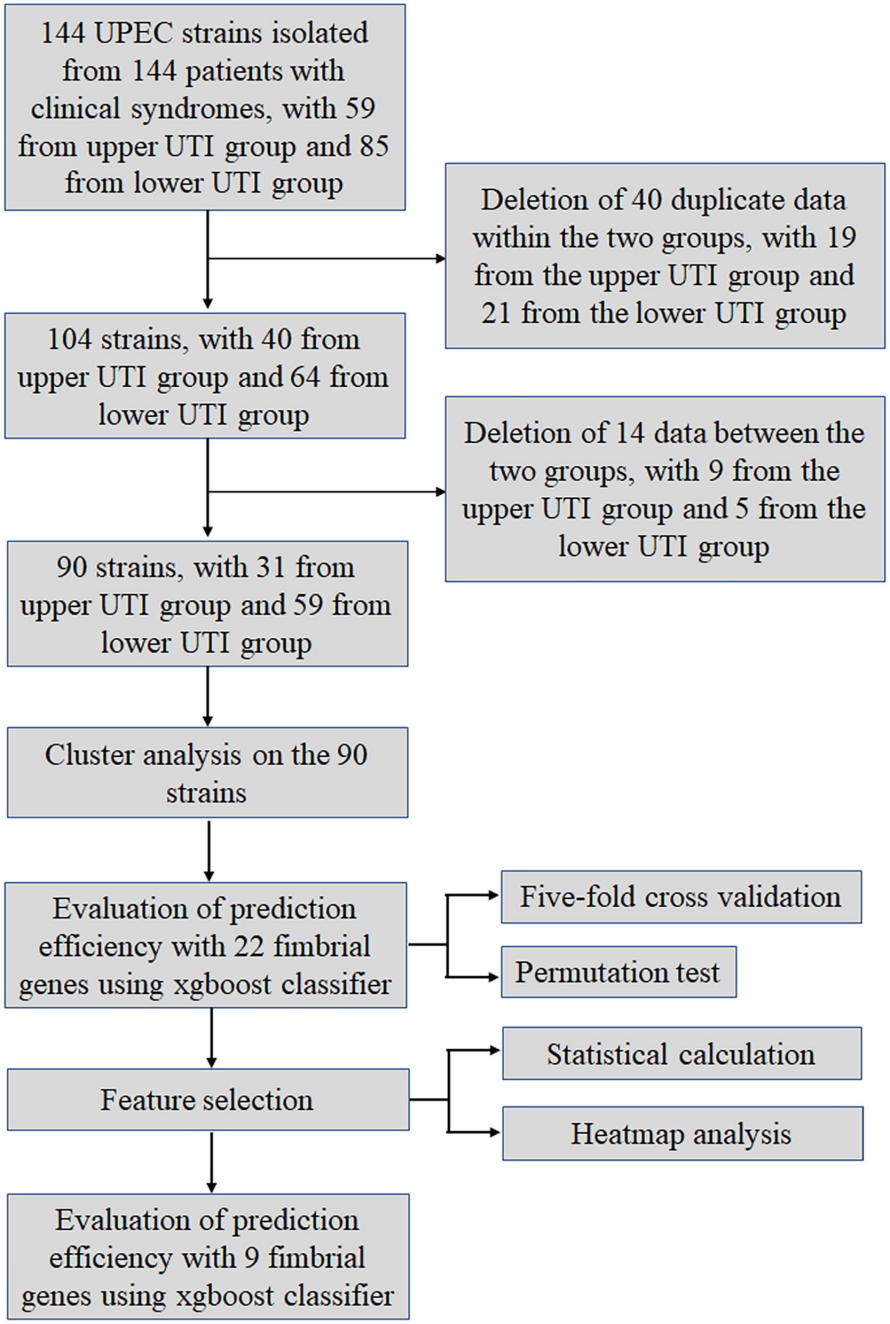
Work flow diagram.

The study design is shown in Figure 1. First, the genomic DNA extracted from the UPEC isolates of patients was examined by PCR for the presence or absence of genes encoding usher proteins of the 22 fimbrial types. The primers are listed in Table 1. Partial agarose gel electrophoresis results are shown in Supplementary Figure 1. The “presence” and “absence” status were regarded as “1” and “0” respectively, in a binary matrix, and the distribution patterns of the 22 fimbrial genes of each strain were changed into a string of numbers (Supplementary Table 1). Therefore, 144 characteristics (number strings) were obtained from the 144 UPEC strains. However, many of the characteristics were duplicated within the upper and lower UTI groups. For example, in the upper UTI group, the No. 3 and No. 5 strains had the same characteristic of “0101111111110001000001upper,” and in the lower UTI group, the No. 88 and No. 90 strains had the same characteristic of “0000011001001001001011lower.” Although the same bacteria were commonly isolated from different patients, the duplicate data could affect the efficiency of the machine learning model. Therefore, a total of 40 strains with the same characteristics were deleted, 19 from the upper UTI group and 21 from the lower UTI group. As a result, 104 UPEC strains remained.

Cluster analysis of the 104 strains was performed using the Euclidean distance and Ward's linkage method (35). The dendrogram of the analysis showed the strains were divided into two groups (Supplementary Figure 2). However, the 104 strains were not completely unique to one of the two groups, that is, some strains in the different groups had the same characteristics, as shown in the red frame. For example, the characteristic of strains No. 7 and No. 63 were shown as “0000011111000001100001upper” and “0000011111000001100001lower,” respectively (Supplementary Table 1). Thus, the distribution of the 22 fimbrial genes was the same, with the only difference that No. 7 came from the upper UTI group, while No. 63 came from the lower UTI group. There were 14 strains from each group that had the same situation. The most likely explanation for this situation was misjudgment by doctors when making the diagnosis. However, although the machine learning model had difficulty analyzing these data, it was not acceptable to simply eliminate all 28 contradictory data sets. Therefore, according to the position of the 28 strains in the dendrogram, five strains were deleted and nine strains were retained in the lower UTI group, while nine strains were deleted and five strains were retained in the upper UTI group. The 14 retained strains are indicated with an asterisk. Thus, 14 strains were deleted, leaving 90 strains for further analysis.

**Figure 2:**
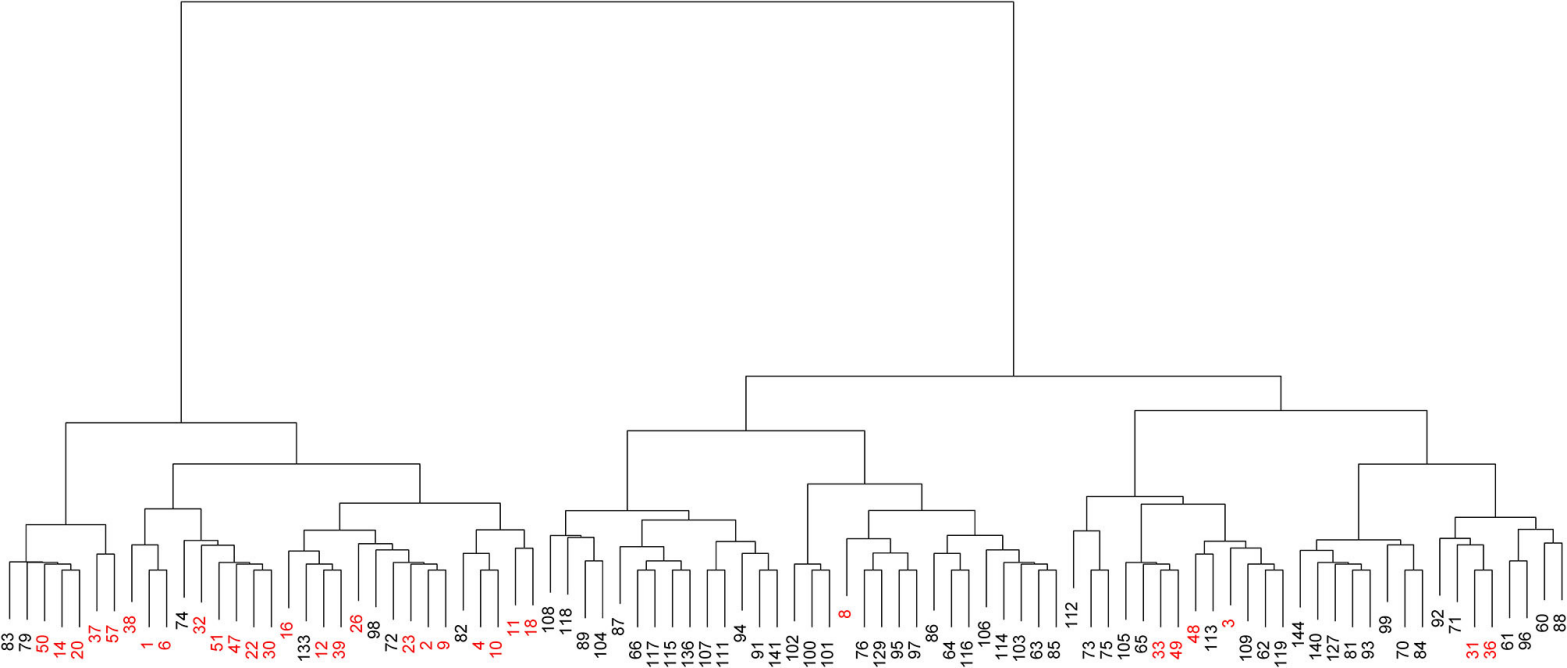
Visualized cluster dendrogram of the distribution patterns of 22 fimbrial genes from 90 strains of uropathogenic *Escherichia coli* (UPEC). Strains from the upper urinary tract infection (UTI) group are shown in red.

According to the similarity between clusters using Ward's method and Euclidean distance, the 90 strains were classified into two groups (Figure 2). The left cluster contained 31 strains, including 24 from the upper UTI group (77.42%, shown in red) and seven from the lower UTI group (22.58%). The right cluster contained the other 59 strains, including 52 from the lower UTI group (88.14%) and seven from the upper UTI group (11.86%, shown in red). Therefore, on the basis of the distribution patterns of the 22 fimbrial genes, the 90 strains were classified into two UTI groups, indicating distribution patterns could be used to predict the infection site.

### Evaluation of Prediction Efficiency With 22 Fimbrial Genes

**Figure 3:**
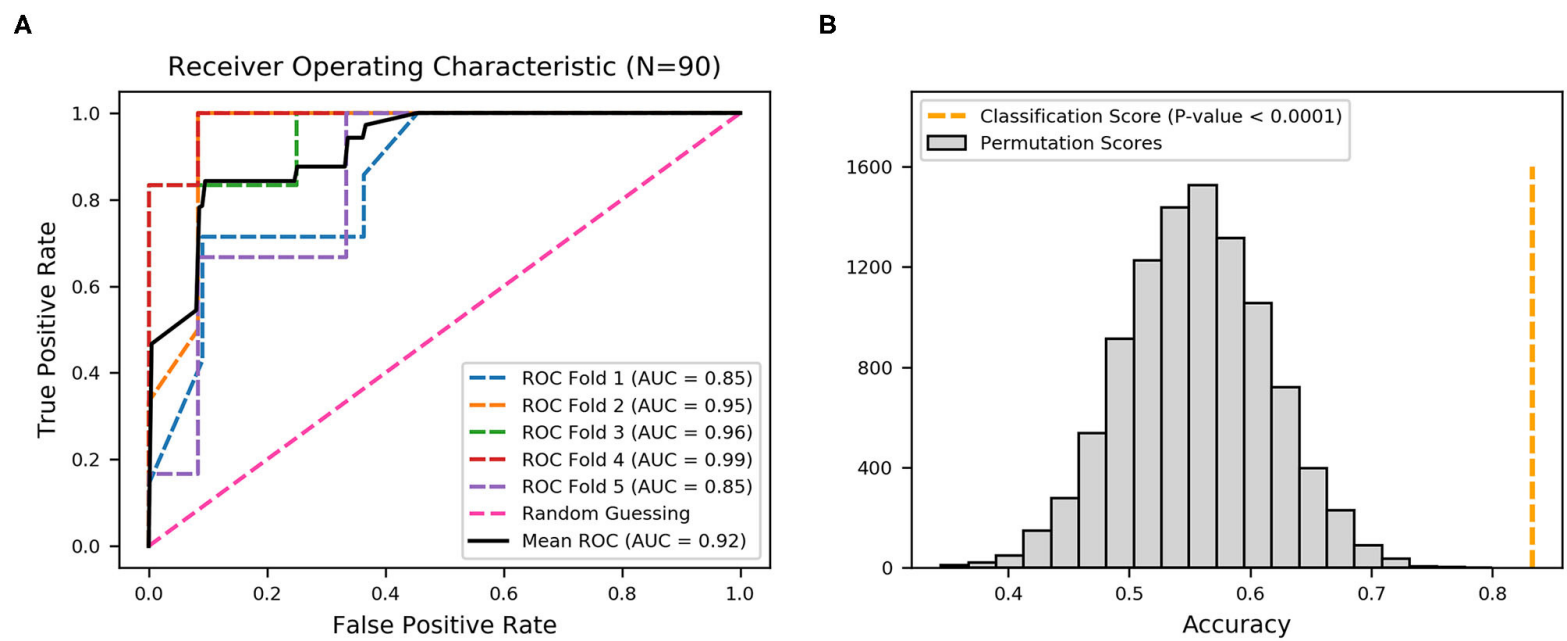
Receiver operating characteristic (ROC) curves produced from 22 fimbrial genes using an XGBoost classifier. **(A)** Five-fold cross-validation to evaluate classifier performance. Area under the curve (AUC) and ROC curve for each fold are presented in different colors, and the average AUC and ROC curve are in black. **(B)** Permutation test (*n* = 10,000) was performed to calculate the statistical significance of the model; the orange dotted line represents the final mean accuracy of the model.

To demonstrate the predictive value, the performance of fimbrial genes distribution for discriminating the infection site was evaluated using machine learning. A model using an extreme gradient boosting algorithm was developed (37), and its predictive performance was assessed using ROC analysis and the AUC of the ROC. The effectiveness was evaluated with 5-fold cross validation from the XGBoost classifier, that is, the 90 sets of data were randomly divided into 5-folds. The ROC curves were averaged for each fold, and final mean AUC and accuracy were calculated. As shown, the XGBoost classifier achieved a mean accuracy of 83.33% and a mean AUC of 0.92 (Figure 3A).

The statistical significance of the analysis was evaluated by permutation test in which the final mean accuracy of the method was compared with an empirical distribution of accuracy values obtained by 10,000 permutations of the random labels. As shown in Figure 3B, the permutation scores were all lower than the classification score (mean accuracy of 83.33%, orange dotted line), with *p*-value < 0.0001, thereby demonstrating high performance in the classification of the derivation of the UPEC strains. Thus, the distribution patterns of the 22 fimbrial genes had the predictive value in identifying upper and lower UTIs.

### Evaluation of Prediction Efficiency After Feature Selection

**Figure 4:**
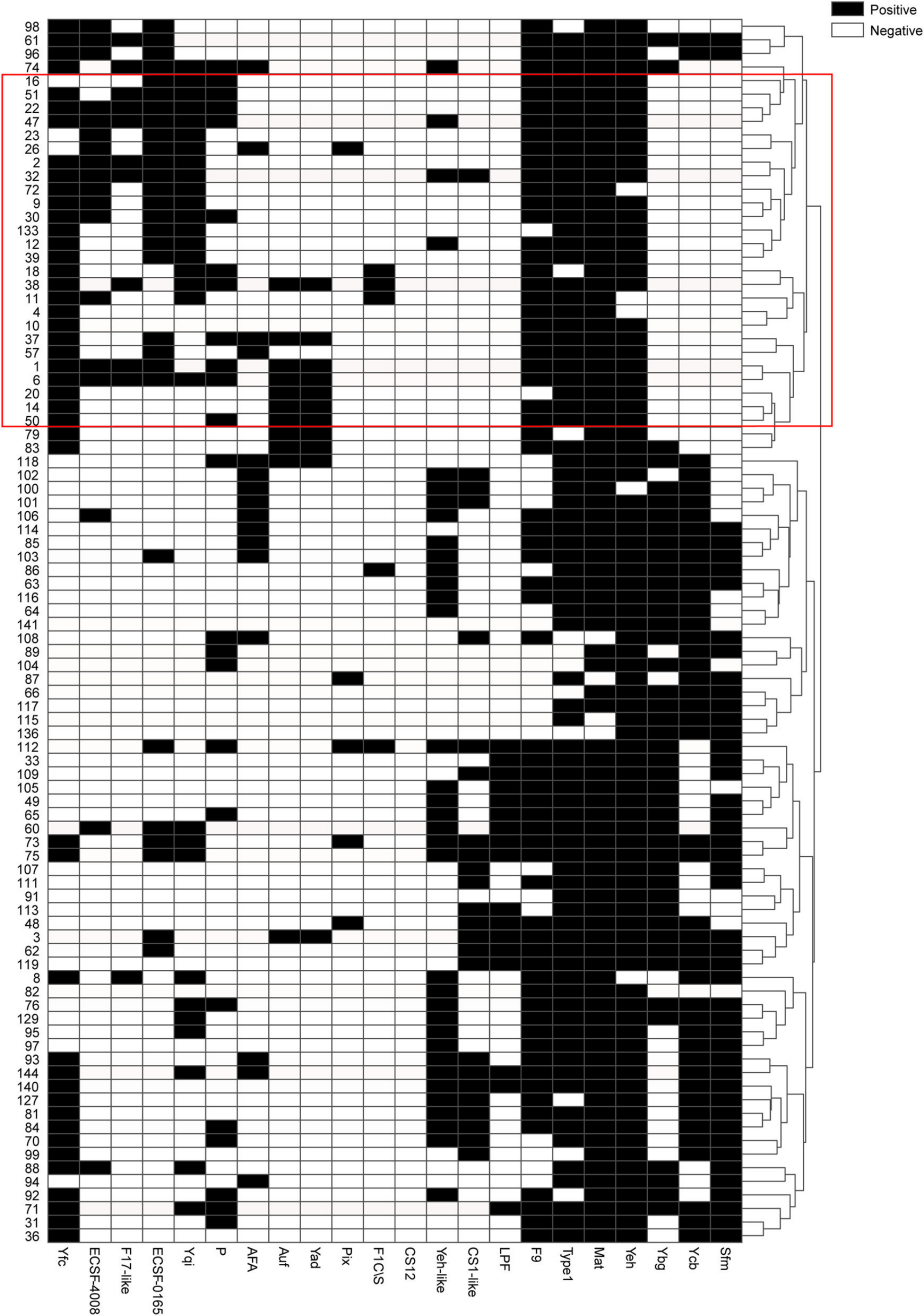
Black and white grid of the distribution patterns of 22 fimbrial genes from 90 strains. Black represents positive status of a gene, whereas white represents negative status of a gene. The 22 fimbrial genes are in columns, and the 90 strains are in rows. The tree on the right shows clustering of the 90 strains, with strains from the upper urinary tract infection group mostly in the red box.

**Figure 5:**
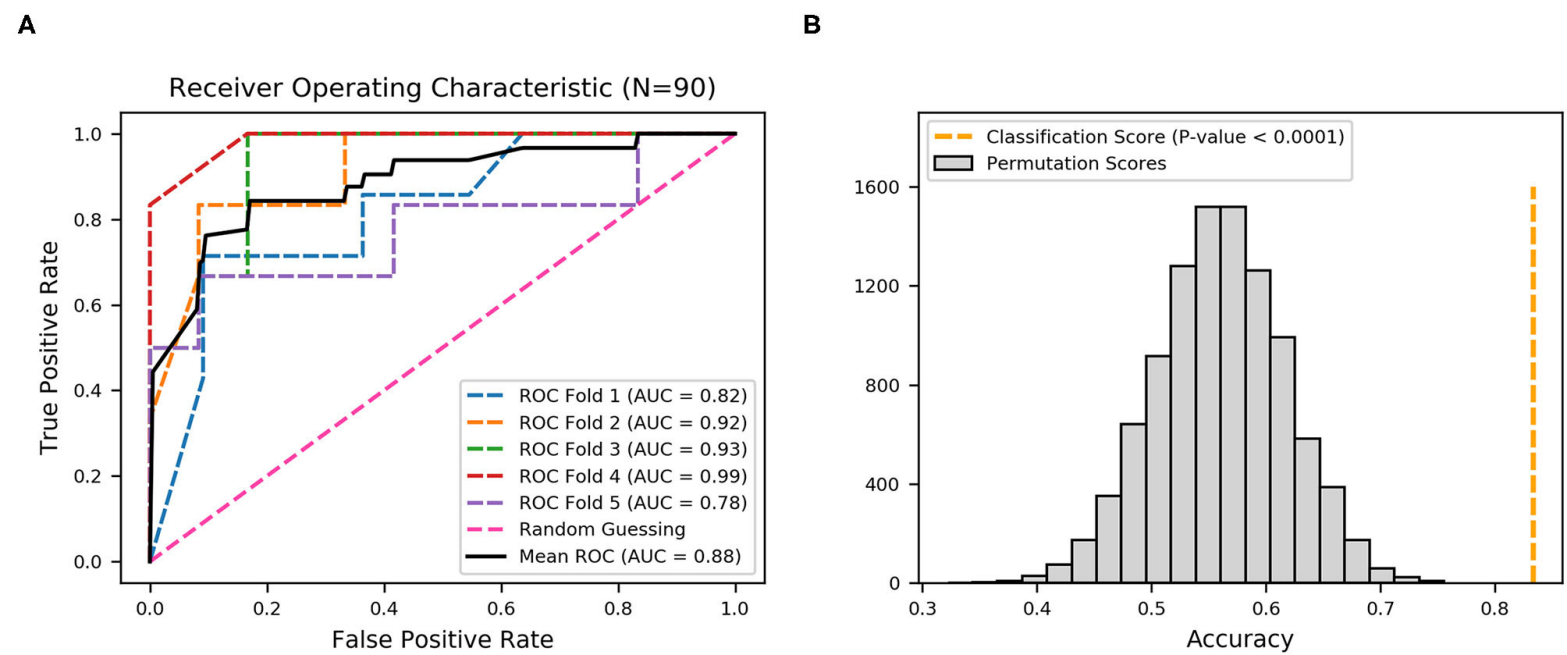
Receiver operating characteristic (ROC) curves produced from nine fimbrial genes after feature selection using XGBoost. **(A)** Five-fold cross-validation to evaluate classifier performance. Area under the curve (AUC) and ROC curve for each fold are in different colors, and the mean AUC and ROC curve are in black. **(B)** Permutation test (*n* = 10,000), with orange dotted line representing the final mean accuracy of the model.

Moreover, the statistical calculation regarding the ratios of different fimbriae and their relevance to the upper and lower UTIs was performed. The significance level was set as *p* < 0.05/22 by Bonferroni correction, and the fimbriae F9, Ycb, Sfm, Yeh-like, Yfc, Ygi, Ybg, ECSF-0165, and F17-like were significantly associated with one of the two groups of UTIs (Table 2). A black and white grid was used to directly display the presence or absence of the 22 fimbrial genes, with black representing positive status of a gene and white representing negative status. The fimbrial genes F9, Yfc, Ygi, ECSF-0165, and F17-like mostly occurred in the upper UTI group, whereas Ycb, Sfm, Yeh-like, and Ybg mostly occurred in the lower UTI group (Figure 4). Then, whether these nine fimbrial genes of the 90 strains could achieve better prediction efficiency than that of the 22 fimbrial genes was evaluated. However, using the distributions of these nine fimbrial genes, the classifier achieved a mean accuracy of 83.33% and a mean AUC of 0.88 (Figure 5). Therefore, the prediction efficiency with the nine genes was similar to and not better than that of the model with 22 fimbrial genes.

**Table 2:** Statistical calculation regarding the ratio of different fimbriae and the relevance to the UTIs.

**Fimbriae**		**Group**		
**Name**	**Status**	**Upper UTI**	**Lower UTI**	***p*-value**
P	Positive	12	12	0.061
	Negative	19	47	
Auf	Positive	8	3	0.007
	Negative	23	56	
F1C/S	Positive	3	2	0.335
	Negative	28	57	
Yad	Positive	8	3	0.007
	Negative	23	56	
CS1-like	Positive	3	21	0.008
	Negative	28	38	
Mat	Positive	31	55	0.294
	Negative	0	4	
Type1	Positive	30	49	0.089
	Negative	1	10	
F9	Positive	30	37	0.000
	Negative	1	22	
Ycb	Positive	5	40	0.000
	Negative	26	19	
Sfm	Positive	6	40	0.000
	Negative	25	19	
LPF	Positive	4	13	0.153
	Negative	27	46	
ECSF-0165	Positive	17	12	0.001
	Negative	14	47	
ECSF-4008	Positive	11	7	0.008
	Negative	20	52	
CS12	Positive	0	0	
	Negative	31	59	
AFA	Positive	3	13	0.145
	Negative	28	46	
Yeh	Positive	28	57	0.335
	Negative	3	2	
Yeh-like	Positive	5	30	0.001
	Negative	26	29	
F17-like	Positive	9	2	0.001
	Negative	22	57	
Yfc	Positive	24	21	0.000
	Negative	7	38	
Pix	Positive	2	3	1
	Negative	29	56	
Ygi	Positive	17	12	0.001
	Negative	14	47	
Ybg	Positive	4	40	0.000
	Negative	27	19	

Thus, the distribution patterns of the 22 fimbrial genes could be used to classify upper and lower UTIs. Among the 22 fimbriae types, F9, Yfc, Ygi, ECSF-0165, and F17-like, as well as Ycb, Sfm, Yeh-like, and Ybg, might be critical in distinguishing between upper and lower UTIs. In addition, the characteristic of CS12 was negative in all 90 strains, indicating that CS12 fimbriae rarely exist in UPEC strains causing UTIs.

## Discussion

Although clinical symptoms can indicate UTIs, only 50–60% of women with dysuria had bacterial UTIs (39). As noted previously, most predictive models for UTIs use a few variables like urine dipstick, urinalysis, or clinical diagnosis. However, there are many limitations. For example, there is currently no consensus accepted level for a positive urine culture with a range from 10^2^ to 10^5^ CFU/mL, and clinical diagnosis maybe relatively subjective. Urinary tract infections are common infectious diseases primarily caused by UPEC, which use fimbriae to adhere to the urethral epithelium, thereby promoting colonization of host cells (40). Although great progress has been made in determining the roles of some fimbriae types in UPEC colonization, there is also little understanding of the roles of other fimbrial types, and whether the combinations of multiple fimbrial types is meaningful in the pathogenesis of UPEC-induced UTIs.

In this study, initially, the hypothesis whether combined multiple fimbrial genes of UPEC could be used to identify the localization of upper and lower UTIs was tested using Ward's method, which is an unsupervised cluster method. Distinct clusters were identified according to the distribution patterns of the 22 fimbrial genes in the UPEC strains. Unsupervised method, however, with lower interpretability, was not suitable to construct a classification model of upper and lower UTIs. By contrast, machine learning based on an XGBoost algorithm is suitable for handling binary classification with stable performance (41, 42). Therefore, a model based on an XGBoost algorithm and multi-fimbriae of UPEC was creatively developed to discriminate upper and lower UTIs. Whereas, the model with 22 fimbrial genes achieved a mean AUC of 0.92 with average accuracy of 83.33%, the model with nine fimbrial genes had a mean AUC of 0.88 with the same average accuracy. Both two model results were statistically significant, demonstrating that distributions of fimbrial genes could discriminate infection sites. Although the AUC of the model with 22 fimbrial genes (AUC = 0.92) was slightly higher than that of the model with nine fimbrial genes (AUC = 0.88), the performance of the model with nine fimbrial genes was similar while also reducing the number of variables after feature selection. In practice, the model with fewer variables may be more easily accepted. Several distinctive features of machine learning, including high mathematical dependence, great classification functioning, and well-behaved fitting, make it superior to traditional methods in the processing of information. Advances in machine learning, coupled with training on large datasets, can improve the accuracy in diagnosing UTIs. However, to be effective, machine learning-based models require further prospective validation. Moreover, empirical antibiotic therapy in UTI treatment results in the abuse of antibiotics and promotes the emergence of multi-drug resistant bacteria. In the future, machine learning could be used to improve drug sensitivity or screen novel antimicrobial targets to develop next-generation therapeutics, and by increasing understanding of the patterns of fimbrial genes, this work could also contribute to the development of fimbriae-based non-antibiotic treatment strategies.

This study also showed that nine fimbriae types might be critical in distinguishing between upper and lower UTIs, that is, F9, Yfc, Ygi, ECSF-0165, and F17-like were mostly in UPEC strains from upper UTIs, while Ycb, Sfm, Yeh-like, and Ybg were mostly in those from the lower UTIs. Among the nine fimbriae types, UPEC F9 fimbriae promote biofilm formation (43), and the tip adhesin FmlH provides a fitness advantage for UPEC colonization of inflamed bladders during chronic cystitis (26); UPEC Ygi fimbriae are necessary for adherence to a kidney cell line, biofilm formation, and *in vivo* fitness in kidneys (29); and F17-like fimbriae promote UPEC intestinal colonization (24); when expressed, *E. coli* K12 *yfc, ycb, sfm*, and *ybg* operons promote adhesion to abiotic and epithelial cell surfaces (44). By contrast, the roles of ECSF-0165 and Yeh-like fimbriae have not yet been determined. In most studies, the occurrence of P fimbriae is 60–90%. For example, 91% (33/35) of the urinary strains causing acute pyelonephritis have P fimbriae (45). However, in the current study, P fimbriae were not well-represented. Three possible reasons could explain their absence: (1) samples were obtained from UTI patients in a single clinical center rather than multiple centers; (2) in the US and Europe, most UPEC strains are from the B2 clade, whereas in East Asia, clade D strains predominate in community-acquired UTIs, followed by B2 strains (46); and (3) previous epidemiological data mainly analyzed the spectrum of infection many years ago, and no recent data are available for comparison. In addition to CU fimbriae, the co-occurrence of other fimbriae families may be meaningful in the identification of infection sites. In the future, research should be conducted on the specific roles and mechanisms of these multiple fimbriae families in the pathogenesis of UTIs. An understanding of the interference or synergy between the adhesion of UPEC fimbriae and other surface adhesins may also provide better targeting therapies for UTI treatment.

Urinary tract infections are currently clinically treated with antibiotics, but the excessive use of antibiotics and the emergence of multi-drug resistant bacteria require urgent development of alternative remedies. The effective alternative remedies to fight UPEC include vaccines, probiotics, estrogens, D-mannose, and D-mannose-derived FimH antagonists (47). D-mannose-derived FimH antagonists can markedly prevent the occurrence of UTIs and recurrent UTIs by selectively depleting intestinal UPEC reservoirs (24). Anti-adhesion strategies are considered to be a promising targeted therapy. Understanding the distribution pattern of fimbriae is fundamental to the development of alternative strategies. Although a completely accurate prediction could not be obtained in this study because of the small sample size, a new method was developed that can potentially identify the site of UTIs, representing an innovative attempt to apply machine learning to predict localization of UTIs. However, the models were built on data from a single hospital within a confined geographic region, and therefore require further validation using strains from other institutions. In addition, the study used symptoms to group the patients and conducted the “machine learning,” while lacking an accurate way to confirm whether a UTI was limited to the lower urinary tract or not. Fresh samples should also be collected for further model validation. In the future, models could be developed using large-scale databases, and machine learning could be employed to accurately predict infection sites using the distribution patterns of 22 fimbrial genes in UPEC strains, which would aid clinicians in their diagnoses, as well as reduce testing cost and improve the quality of life.

## Data Availability Statement

The original contributions presented in the study are included in the article/Supplementary Material, further inquiries can be directed to the corresponding author.

## Ethics Statement

The studies involving human participants were reviewed and approved by the Ethics Committee of Tianjin Medical University. The patients/participants provided their written informed consent to participate in this study.

## Author Contributions

XL wrote the main manuscript text. KZ, JW, JG, JR, TG, WS, and MZ performed the experiments. YC provided the UPEC strains. ZY supervised the experimental framework. QW supervised the analyses. All authors have read and agreed to the published version of the manuscript.

## Conflict of Interest

The authors declare that the research was conducted in the absence of any commercial or financial relationships that could be construed as a potential conflict of interest.
