# Supplementary material for: Diagnostic Value of the Fimbriae Distribution Pattern in Localization of Urinary Tract Infection
Source: Front Med (Lausanne). 2021 Jun 18;8:602691. doi: 10.3389/fmed.2021.602691 (PMC8249706; doi:10.3389/fmed.2021.602691)
Supplement: Supplementary file 1 [file Data_Sheet_1.docx]

Supplementary Material

## Supplementary Figures


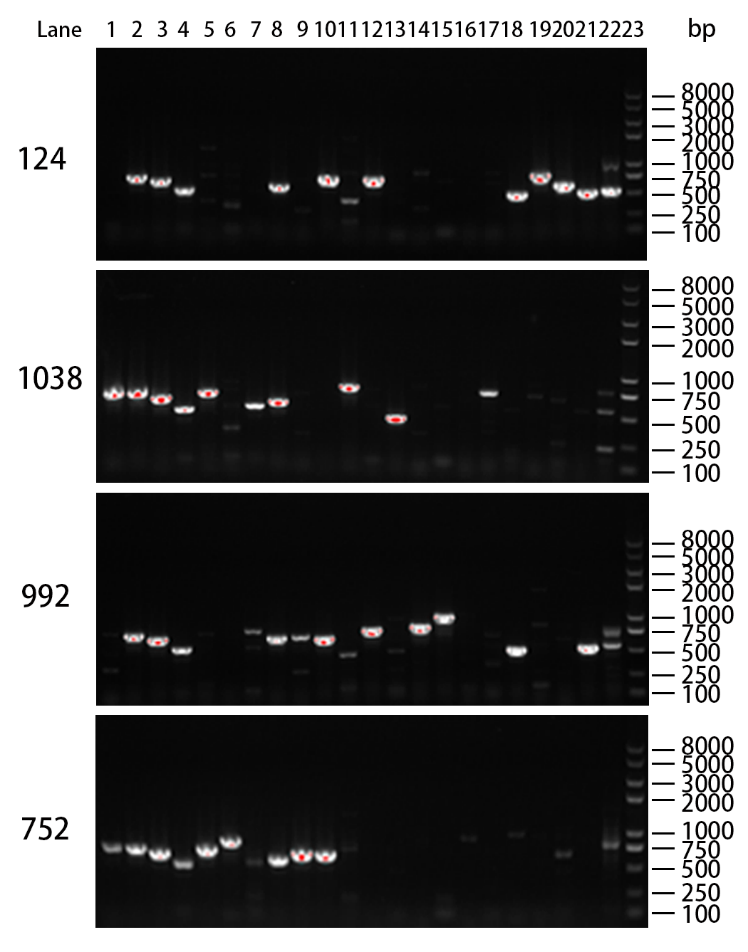


**Supplementary Figure 1.** Agarose gel electrophoresis followed by amplification of 22 fimbrial genes. Lanes 1to 22 are respectively the following 22 fimbrial usher protein encoding genes: CS1-like, Mat, Type 1, F9, Ycb, Sfm, LPF, Yeh, Yeh-like, Yfc, Pix, Yqi, Ybg, ECSF-0165, ECSF-4008, CS12, Afa, F17-like, Auf, F1C/S, P and Yad. Lane 23 is a DNA ladder consisting of DNA fragments 8,000 base pairs in length (Trans Gen Biotech, Beijing, China). The amplifications were from four genomes. The amplicon size of each gene is shown in Table 1.


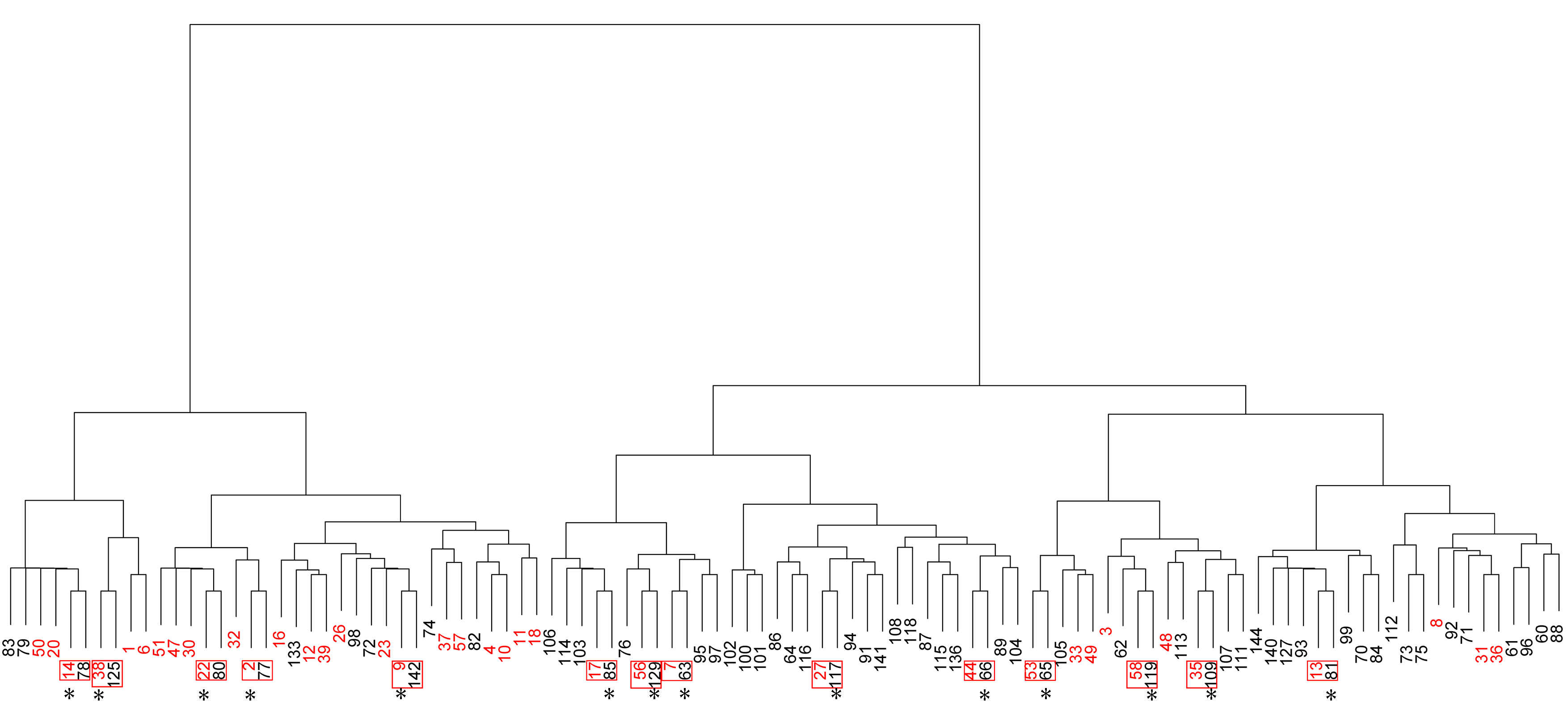


**Supplementary Figure 2.** Cluster dendrogram of the 104 uropathogenic *Escherichia coli* (UPEC) strains. The number in red represents strains from the upper urinary tract infection (UTI) group, and the red frame represents the duplicated data between upper and lower groups. The strains with asterisks are the five strains retained in the upper UTI group and the nine strains retained in the lower UTI group.

## Supplementary Tables

**Table S1. 144 Bacterial strains and the Characteristic s of the 22 fimbrial genes.**

| **Strain** | **Sex** | **Age** | **Group** | **Characteristic*** | **Description** |
| --- | --- | --- | --- | --- | --- |
| **1** | Female | 63 | Upper | 1101011100011001011000upper |  |
| **2** | Male | 61 | Upper | 0000011100011001011010upper | b |
| **3** | Male | 57 | Upper | 0101111111110001000001upper | a |
| **4** | Male | 61 | Upper | 0000011100000000001000upper |  |
| **5** | Male | 82 | Upper | 0101111111110001000001upper | a, # |
| **6** | Male | 67 | Upper | 1101011100011001011010upper |  |
| **7** | Male | 82 | Upper | 0000011111000001100001upper | a, b, # |
| **8** | Male | 69 | Upper | 0000011111000000111010upper |  |
| **9** | Female | 57 | Upper | 0000011100011001001010upper | a, b |
| **10** | Female | 55 | Upper | 0000011100000001001000upper | a |
| **11** | Male | 72 | Upper | 0010011100001000001010upper |  |
| **12** | Female | 29 | Upper | 0000011100010001101010upper |  |
| **13** | Male | 62 | Upper | 0000111111000001101000upper | a, b, # |
| **14** | Female | 86 | Upper | 0101011100000001001000upper | a, b |
| **15** | Female | 69 | Upper | 0101011100000001001000upper | a, # |
| **16** | Male | 79 | Upper | 1000011100010001000010upper |  |
| **17** | Female | 33 | Upper | 0000011111000011100001upper | a, b, # |
| **18** | Male | 58 | Upper | 1010010100000001001010upper |  |
| **19** | Male | 77 | Upper | 0000011111000011100001upper | a, # |
| **20** | Male | 64 | Upper | 0101011000000001001000upper |  |
| **21** | Female | 63 | Upper | 0101011100000001001000upper | a, # |
| **22** | Female | 55 | Upper | 1000011100011001011010upper | b |
| **23** | Female | 48 | Upper | 0000011100011001000010upper |  |
| **24** | Female | 90 | Upper | 0000011111000001100001upper | a, # |
| **25** | Male | 77 | Upper | 0000011111000011100001upper | a, # |
| **26** | Female | 92 | Upper | 0000011100011011000110upper |  |
| **27** | Female | 30 | Upper | 0000011011000001000001upper | b, # |
| **28** | Female | 54 | Upper | 0101011100000001001000upper | a, # |
| **29** | Male | 52 | Upper | 0000011111000001100001upper | a, # |
| **30** | Female | 70 | Upper | 1000011100011001001010upper | a |
| **31** | Male | 61 | Upper | 1000011111000001001000upper |  |
| **32** | Female | 34 | Upper | 0000111100011001111010upper |  |
| **33** | Female | 69 | Upper | 0000011101100001000001upper |  |
| **34** | Female | 76 | Upper | 0000011100000001001000upper | a, # |
| **35** | Female | 40 | Upper | 0000111101100001000001upper | b, # |
| **36** | Female | 65 | Upper | 0000011111000001001000upper |  |
| **37** | Female | 55 | Upper | 1101011100010011001000upper |  |
| **38** | Male | 83 | Upper | 1111011100000001011010upper | b |
| **39** | Female | 54 | Upper | 0000011100010001001010upper | a |
| **40** | Female | 64 | Upper | 0101011100000001001000upper | a, # |
| **41** | Female | 62 | Upper | 0101011100000001001000upper | a, # |
| **42** | Male | 64 | Upper | 1000011100011001001010upper | a, # |
| **43** | Male | 64 | Upper | 0101011100000001001000upper | a, # |
| **44** | Female | 76 | Upper | 0000010011000001000001upper | a, b, # |
| **45** | Female | 40 | Upper | 0000111111000001101000upper | a, # |
| **46** | Female | 76 | Upper | 0000010011000001000001upper | a, # |
| **47** | Male | 67 | Upper | 1000011100011001111010upper |  |
| **48** | Female | 59 | Upper | 0000111110100001000101upper |  |
| **49** | Female | 65 | Upper | 0000011101100001100001upper |  |
| **50** | Female | 55 | Upper | 1101011100000001001000upper |  |
| **51** | Male | 66 | Upper | 1000011100010001011010upper |  |
| **52** | Female | 69 | Upper | 0000011100011001001010upper | a, # |
| **53** | Male | 61 | Upper | 1000011101100001100001upper | b, # |
| **54** | Female | 74 | Upper | 0000011111000011100001upper | a, # |
| **55** | Female | 56 | Upper | 0000011100010001001010upper | a, # |
| **56** | Female | 68 | Upper | 0000011111000001100011upper | b, # |
| **57** | Female | 28 | Upper | 0000011100010011001000upper |  |
| **58** | Male | 83 | Upper | 0000111111100001000001upper | b, # |
| **59** | Male | 63 | Upper | 0000011111000001100001upper | a, # |
| **60** | Male | 68 | Lower | 0000011101111001100011lower |  |
| **61** | Male | 49 | Lower | 0000011111011001011001lower |  |
| **62** | Female | 81 | Lower | 0000111111110001000001lower |  |
| **63** | Female | 72 | Lower | 0000011111000001100001lower | a, b |
| **64** | Male | 78 | Lower | 0000011010000001100001lower |  |
| **65** | Female | 51 | Lower | 1000011101100001100001lower | b |
| **66** | Male | 65 | Lower | 0000010011000001000001lower | a, b |
| **67** | Male | 49 | Lower | 0000011111000001100001lower | a, # |
| **68** | Female | 61 | Lower | 0000011111000001100001lower | a, # |
| **69** | Female | 92 | Lower | 0000011111000001100001lower | a, # |
| **70** | Female | 71 | Lower | 1000111011000001101000lower |  |
| **71** | Male | 64 | Lower | 1000011111100001001011lower |  |
| **72** | Female | 69 | Lower | 0000011100011000001010lower |  |
| **73** | Male | 85 | Lower | 0000111111110001101111lower |  |
| **74** | Male | 53 | Lower | 1000011100010011111011lower |  |
| **75** | Male | 87 | Lower | 0000111111110001101011lower |  |
| **76** | Male | 72 | Lower | 1000011111000001100011lower |  |
| **77** | Female | 86 | Lower | 0000011100011001011010lower | b, # |
| **78** | Female | 79 | Lower | 0101011100000001001000lower | a, b, # |
| **79** | Female | 102 | Lower | 0101010100000001001000lower | a |
| **80** | Female | 65 | Lower | 1000011100011001011010lower | a, b, # |
| **81** | Male | 85 | Lower | 0000111111000001101000lower | b |
| **82** | Female | 59 | Lower | 0000011100000001100000lower |  |
| **83** | Female | 73 | Lower | 0101011100000001001001lower |  |
| **84** | Female | 53 | Lower | 1000111111000001101000lower |  |
| **85** | Female | 74 | Lower | 0000011111000011100001lower | a, b |
| **86** | Female | 87 | Lower | 0010011011000001100001lower |  |
| **87** | Male | 65 | Lower | 0000001011000001000100lower |  |
| **88** | Female | 86 | Lower | 0000011001001001001011lower | a |
| **89** | Female | 75 | Lower | 1000010011000001000000lower |  |
| **90** | Female | 83 | Lower | 0000011001001001001011lower | a, # |
| **91** | Female | 67 | Lower | 0000011000000001000001lower | a |
| **92** | Male | 68 | Lower | 1000010101000001101001lower |  |
| **93** | Male | 25 | Lower | 0000111111000011101000lower |  |
| **94** | Female | 60 | Lower | 0000011001000011000001lower | a |
| **95** | Female | 79 | Lower | 0000011111000001100010lower |  |
| **96** | Female | 70 | Lower | 0000011111011001001000lower |  |
| **97** | Male | 82 | Lower | 0000011111000001100000lower |  |
| **98** | Male | 80 | Lower | 0000010100011001001000lower |  |
| **99** | Female | 61 | Lower | 0000110011000001001000lower |  |
| **100** | Male | 65 | Lower | 0000111010000010100001lower |  |
| **101** | Female | 51 | Lower | 0000111010000011100001lower |  |
| **102** | Male | 83 | Lower | 0000111010000011100000lower |  |
| **103** | Female | 39 | Lower | 0000011111010011100001lower |  |
| **104** | Male | 72 | Lower | 1000010010000001000001lower |  |
| **105** | Female | 72 | Lower | 0000011100100001100001lower |  |
| **106** | Female | 72 | Lower | 0000011110001011100001lower |  |
| **107** | Female | 72 | Lower | 0000111001000001000001lower |  |
| **108** | Male | 84 | Lower | 1000100111000011000001lower |  |
| **109** | Female | 88 | Lower | 0000111101100001000001lower | b |
| **110** | Female | 66 | Lower | 0000011001000011000001lower | a, # |
| **111** | Female | 55 | Lower | 0000111101000001000001lower |  |
| **112** | Male | 73 | Lower | 1010111101110001100101lower |  |
| **113** | Female | 68 | Lower | 0000111000100001000001lower |  |
| **114** | Male | 71 | Lower | 0000011111000011000001lower |  |
| **115** | Female | 61 | Lower | 0000001011000001000001lower | a |
| **116** | Male | 67 | Lower | 0000011110000001100001lower |  |
| **117** | Female | 49 | Lower | 0000011011000001000001lower | a, b |
| **118** | Female | 87 | Lower | 1101011010000011000001lower |  |
| **119** | Female | 36 | Lower | 0000111111100001000001lower | a, b |
| **120** | Female | 58 | Lower | 1000011100011001011010lower | a, # |
| **121** | Male | 80 | Lower | 0000011011000001000001lower | a, # |
| **122** | Male | 67 | Lower | 0000111111100001000001lower | a, # |
| **123** | Female | 60 | Lower | 0000111111100001000001lower | a, # |
| **124** | Female | 53 | Lower | 0101011100000001001000lower | a, # |
| **125** | Male | 83 | Lower | 1111011100000001011010lower | a, b, # |
| **126** | Male | 63 | Lower | 1111011100000001011010lower | a, # |
| **127** | Female | 66 | Lower | 0000110111000001101000lower |  |
| **128** | Male | 83 | Lower | 0000010011000001000001lower | a, # |
| **129** | Male | 57 | Lower | 0000011111000001100011lower | b |
| **130** | Male | 80 | Lower | 0000011000000001000001lower | a, # |
| **131** | Female | 54 | Lower | 0000011111000011100001lower | a, # |
| **132** | Female | 84 | Lower | 0000001011000001000001lower | a, # |
| **133** | Female | 64 | Lower | 0000011000010001001010lower |  |
| **134** | Female | 44 | Lower | 0101010100000001001000lower | a, # |
| **135** | Male | 79 | Lower | 1111011100000001011010lower | a, # |
| **136** | Female | 76 | Lower | 0000000011000001000001lower |  |
| **137** | Male | 76 | Lower | 0000011111000011100001lower | a, # |
| **138** | Male | 82 | Lower | 1000011100011001011010lower | a, # |
| **139** | Male | 60 | Lower | 0000011111000001100001lower | a, # |
| **140** | Female | 83 | Lower | 0000111111100001101000lower |  |
| **141** | Female | 45 | Lower | 0000011010000001000001lower |  |
| **142** | Male | 94 | Lower | 0000011100011001001010lower | b, # |
| **143** | Female | 90 | Lower | 0101011100000001001000lower | a, # |
| **144** | Female | 89 | Lower | 0000111111100011101010lower |  |

*: The string of numbers shown as the order of P, Auf, F1C/S, Yad, CS1-like, Mat, Type 1, F9, Ycb, Sfm, LPF, ECSF-0165, ECSF-4008, CS12, Afa, Yeh, Yeh-like, F17-like, Yfc, Pix, Yqi, and Ybg.

a: The duplicated data within the upper and lower group respectively.

a, #: The deleted duplicated data within the upper and lower group respectively.

b: The duplicated data between upper and lower group.

b, #: The deleted duplicated data between upper and lower group.
